# Real-world insights into genomic reprogramming during CAR-T cell manufacturing in India

**DOI:** 10.3389/fimmu.2026.1912690

**Published:** 2026-09-03

**Authors:** Nupur Das, Prashant Mehta, Kusum Gupta, Rahul Katharia, Swati Pabbi, Pravas Mishra, Soni Morya

**Affiliations:** 1 Department of Pathology, School of Medicine, Amrita Vishwa Vidyapeetham, Faridabad, Haryana, India; 2 Department of Medical Oncology, School of Medicine, Amrita Vishwa Vidyapeetham, Faridabad, Haryana, India; 3 Department of Transfusion Medicine, School of Medicine, Amrita Vishwa Vidyapeetham, Faridabad, Haryana, India

**Keywords:** CAR-T, genomic profiling, immune cells, leukapheresis, NanoString, reactome pathway

## Abstract

**Background:**

While CAR-T cell therapy has transformed outcomes in B-cell malignancies, most genomic insights originate from clinical trials. There is a paucity of data describing molecular changes during CAR-T manufacturing in real-world practice, particularly for non-US CAR constructs.

**Methods:**

This was an observational study conducted at a tertiary care center in India where a total of 4 set of paired samples comprising leukapheresis starting material (n=4) and the corresponding final CAR-T cell product (n=4) were analyzed. Analysis was done using NanoString nCounter^®^ platform with a targeted immune-oncology panel comprising 750 genes.

**Results:**

Comparative gene expression analysis between CAR-T product and leukapheresis sample revealed significant upregulation of genes associated with cell proliferation (MKI67, BUB1), cytokine responsiveness (IL2RA, IL12RB2, CISH), metabolic fitness (PHGDH, PSAT1), and effector differentiation (IRF4, BATF3, LIF) in final product as compared to leukapheresis sample. Concurrent downregulation of innate immune and myeloid lineage genes (CD14, FCGR3A/B, FCAR, CYBB, FPR1, LILRA5, TYROBP, S100A12) along with reduced expression of inflammatory mediators (FOS, DUSP1, S100A12) suggests a controlled activation state that may limit baseline inflammatory priming and effective enrichment of adaptive T cells.

**Conclusions:**

Indigenous CAR-T manufacturing processes induce significant transcriptomic remodeling and reflects a highly proliferative and cytokine-responsive CAR-T phenotype associated with improved functional fitness. These findings may reflect a baseline framework for CAR-T genomic characterization in real-world clinical settings to explore outcome-related analyses.

## Introduction

Chimeric antigen receptor (CAR)-T cell therapy has emerged as a paradigm-shifting immunotherapeutic option with durable responses in earlier non-responsive or relapsed B-cell and plasma cell neoplasms (1). Recent innovations in CAR construct design, manufacturing processes and immune engineering are accelerating the translation of CAR-T therapy to solid tumors while simultaneously improving the safety, efficacy and accessibility of next-generation and indigenous CAR-T platforms (2). Despite the remarkable clinical success of CD19-directed CAR-T cell therapy in relapsed or refractory B-cell malignancies, its widespread implementation remains constrained by multiple challenges including complex manufacturing processes, high treatment costs, limited accessibility, product heterogeneity and variable CAR-T cell persistence and durability (3). Nonetheless, the treatment outcomes remain heterogenous and variable across different clinical phenotypes of B-lymphoid malignancies. Post-infusion CAR T-cell efficacy and toxicity is determined by interindividual variability in expansion, persistence, response, and toxicity profile. This highlights the importance of both host-dependent and manufacturing-dependent intrinsic T-cell biology in CAR-T manufacture. Emerging evidence indicates that pre-infusion product characteristics can predict clinical outcomes (4). Thus, comparing baseline cellular profile at leukapheresis with the final CAR-T product may provide critical insights into manufacturing-driven molecular remodeling. Most available data, however, focuses on US based CAR-T products (axicabtagene ciloleucel, tisagenlecleucel and lisocabtagene maraleucel) evaluated within controlled trial frameworks (5, 6).

Low- and middle-income countries (LMICs) face a significant challenge with regard to CAR-T cell therapy access. In India, CAR-T therapy has recently entered routine clinical practice with the development of indigenous CD19 CAR-T products viz Talicabtagene autoleucel (indigenous, Humanized, HCAR-19) and Varnimcabtagene autoleucel (manufactured in India using CliniMACS, Prodigy). Growing clinical use of Indian CAR-T represents a major milestone in expanding access to advanced cellular therapies with the cost being 1/10th of US counterparts. While early clinical outcomes have been encouraging, there are currently no published studies providing deep genomic or transcriptomic characterization of CAR-T cell products manufactured and administered in India. This lack of molecular data limits biological benchmarking, cross-platform comparability, and the development of locally relevant predictive biomarkers.

Herein, paired transcriptomic profiling of leukapheresis samples and corresponding final CAR T-cell products were done. Using a targeted NanoString™ immune-oncology gene panel comprising approximately 750 genes, we aimed to define core genomic programs induced during CAR-T cell manufacturing in routine clinical practice in India.

## Materials and methods

This is an exploratory, observational study conducted at a tertiary care academic center in India. Four patients with relapsed/refractory B-cell malignancies (B-ALL & DLBCL) undergoing treatment with CAR-T cell therapy were included. For each patient, paired samples comprising leukapheresis starting material and the corresponding final CAR-T cell product were analyzed. The study was approved by the institutional ethics committee, and written informed consent was obtained from all four patients in accordance with the Declaration of Helsinki.

### Leukapheresis and CAR-T cell manufacturing

Peripheral blood mononuclear cells were collected by leukapheresis according to manufacturer approved standard operating procedures. CAR T-cell products were manufactured by the manufacturer using standard approved and validated clinical manufacturing protocol. Final CAR T-cell products were sampled from residual left over product after infusion from the CAR-T bag.

Leukapheresis for chimeric antigen receptor (CAR) T-cell manufacture was performed using the Spectra Optia apheresis system (Terumo BCT). Peripheral blood lymphocytes were harvested from non-mobilized patients in accordance with the standard operating procedures and guidelines. The target absolute lymphocyte count (ALC) of the leukapheresis product was pre-defined as 2 × 10^9^ lymphocytes as per manufacturer’s recommended criteria. The mononuclear cell (MNC) collection protocol was individualized based on patient weight, pre-procedure absolute lymphocyte count and haematocrit. The procedures were performed using central venous access. Acid citrate dextrose solution A (ACD-A) was used as an anticoagulant at a ratio of 1:12 with calcium supplementation prophylactically to mitigate citrate-related toxicity. Leukapheresis products were transported under controlled conditions to the manufacturing facility for T-cell selection, activation, genetic modification, and expansion.

### Characterization of leukapheresis and CAR-T bag/product

Prior to CAR-T cell manufacturing, leukapheresis product were characterized using an automated hematology analyzer (ADVIA 2120i, Siemens Healthineers) to determine total leucocyte count and leukocyte differential including neutrophils, lymphocytes, monocytes, eosinophils and basophils (**Supplementary Table 1**). Immunophenotypic characterization of CAR-T bag/product was performed by multiparametric flow cytometry using fluorochrome-conjugated monoclonal antibodies against CD3, CD4, CD8, CD45 and CD19 (**Supplementary Figure 1**). The frequency of total T cells (CD3^+^), CD4^+^ helper T cells, CD8^+^ cytotoxic T cells, CD4:CD8 ratio and B cells was determined according to a predefined gating strategy (**Supplementary Table 2**). These analyses were performed to characterize the cellular composition of the input leukapheresis material and the manufactured CAR-T bag/product, thereby providing phenotypic context for the downstream transcriptomic and functional analyses.

### RNA extraction and NanoString gene expression profiling

Total RNA was extracted from leukapheresis samples and final CAR T-cell products using standardized extraction protocols (**Supplementary Data**). Cryopreserved CAR-T bag/product were thawed according to institutional standard operating procedures and washed with phosphate-buffered saline (PBS) containing 2% fetal bovine serum to remove residual cryoprotectant (DMSO). Following centrifugation, cells were immediately resuspended and processed for RNA extraction without any ex vivo stimulation or culture expansion. Cell viability was assessed immediately after thawing, and only samples fulfilling predefined quality criteria were included in downstream analyses. The interval between completion of washing and sample processing was minimized (approximately 60–90 minutes) and all samples were handled using an identical standardized workflow to reduce technical variability. RNA quantity and quality were assessed prior to downstream analysis. Gene expression profiling was performed using the NanoString nCounter^®^ platform with a targeted immune-oncology panel comprising approximately 750 genes. This panel was designed to capture key biological pathways relevant to CAR T-cell function, including immune activation, cytotoxicity, T-cell differentiation, exhaustion, metabolism, proliferation, and cytokine signaling. Hybridization, post-hybridization processing, and digital counting were performed according to the manufacturer’s instructions.

### Data processing and statistical analysis

Raw NanoString counts were processed using NanoString nSolver™ 4.0 analysis software (NanoString Technologies, Seattle, WA) following the manufacturer’s recommended workflow. Quality control metrics were assessed for all samples, and background subtraction was performed using negative control probes. Raw transcript counts were normalized using internal positive controls and a panel of 10 housekeeping genes through the advanced analysis plugin (version 2.0.115).

**Figure 1:**
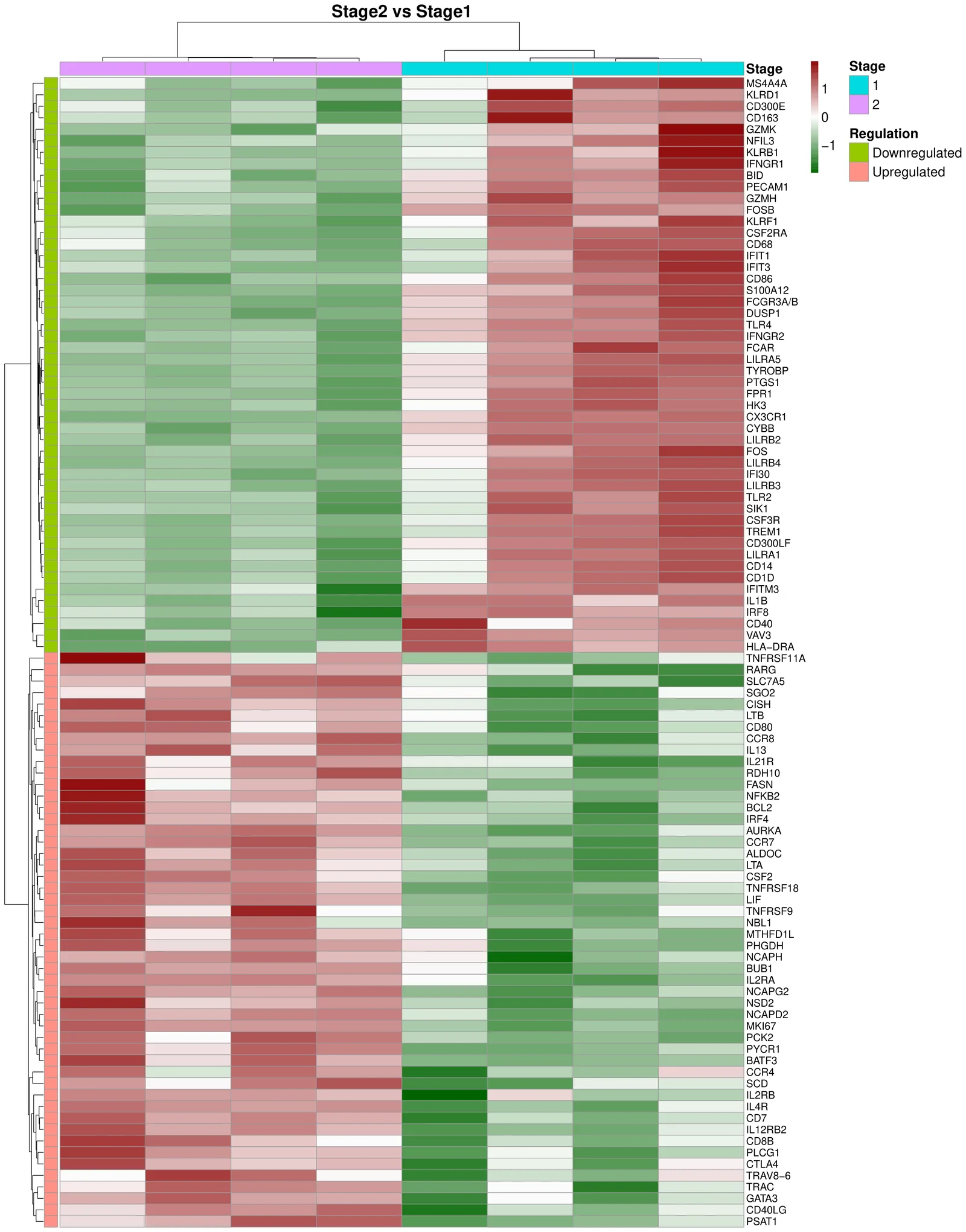
Heatmap of the top 100 differentially expressed genes identified by NanoString transcriptomic profiling comparing Stage 1 (leukapheresis) and Stage 2 (manufactured CAR-T bag/product) samples. Rows represent individual genes and columns represent study samples. Hierarchical clustering was performed using normalized gene expression values; (Red indicates relatively higher expression and green indicates relatively lower expression, as shown in the accompanying color scale).

Given the paired study design, differential gene expression analysis was performed by comparing each leukapheresis sample with its matched CAR-T cell bag/product. Fold changes in gene expression were calculated based on sample type, and statistical significance was determined using a paired two-tailed t-test implemented within the nSolver advanced analysis module. To account for multiple comparisons across the NanoString immune gene panel, Benjamini–Hochberg false discovery rate (FDR) correction was applied. Genes were considered differentially expressed if they met the criteria of an FDR-adjusted *P* value < 0.05 and an absolute fold change ≥1.5.

(Upregulated, fold change >1.5; downregulated, fold change <−1.5). Differentially expressed genes were subsequently used for downstream pathway enrichment analyses. Results: Global transcriptomic analysis demonstrated clear separation between leukapheresis samples and final CAR-T products, indicating substantial manufacturing-associated molecular divergence. Comparative gene expression analysis between the CAR-T bag/product sample and the leukapheresis sample revealed distinct transcriptional changes as shown in the heat map in **Figure 1**.

### Significantly upregulated genes in CAR-T product

**Figure 2:**
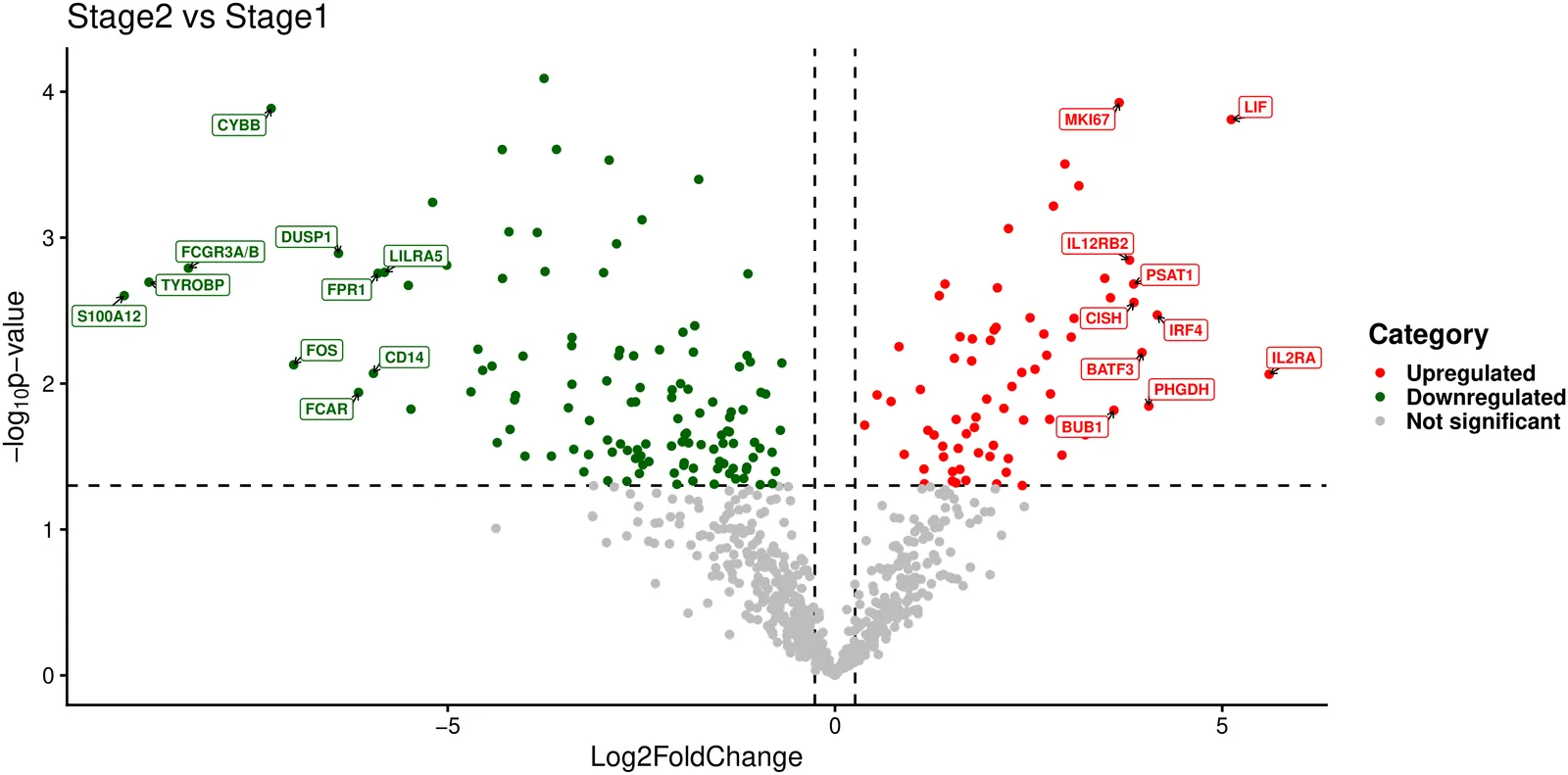
Volcano plot illustrating differential gene expression between manufactured CAR-T bag/product (Stage 2) and leukapheresis (Stage 1) samples. Each point represents an individual gene, plotted according to log_2_ fold change (x-axis) and −log_10_ (P value) (y-axis). Significantly upregulated genes are shown in red, significantly downregulated genes in green, and non-significant genes in gray. The horizontal dashed line denotes the statistical significance threshold.

The CAR-T product showed marked upregulation of genes linked to cell cycle progression, cytokine signaling, transcriptional activation and metabolic reprogramming, indicating successful ex vivo activation and expansion (**Table 1**). **Figure 2** shows top ten significantly upregulated and downregulated genes. Genes which are upregulated include:

**Table 1:** Top upregulated and downregulated genes in CAR-T product compared to Leukapheresis sample.

Category	Gene/pathway	Fold change in CAR-T product	Functional annotation	Interpretation in CAR-T context	Clinical relevance
Gene	MKI67	+12.72	Proliferation marker	Active clonal expansion	Expansion potency
Gene	BUB1	+ 12.14	Mitotic checkpoint kinase	Cell cycle progression	Proliferative fitness
Gene	IL2RA (CD25)	+48.69	IL-2 receptor	Cytokine responsiveness	expansion & persistence
Gene	IL12RB2	+13.95	Th1 receptor	Cytotoxic polarization	Effector function
Gene	CISH	+14.52	cytokine feedback	Controlled cytokine signaling	Activation regulation
Gene	IRF4	+17.89	activation TF	Effector differentiation	CAR-T functionality
Gene	BATF3	+15.6	differentiation TF	Effector programming	Cytotoxic competence
Gene	PHGDH	+16.58	serine metabolism	Metabolic adaptation	Persistence potential
Gene	PSAT1	+14.49	amino acid synthesis	Biosynthetic support	Proliferative capacity
Gene	LIF	+34.68	gp130 cytokine	Survival signaling	Durability *in vivo*
Gene	CD14	-62.22	monocyte marker	Reduced myeloid cells	Product purity
Gene	FCGR3A/B	-325.83	Fc receptor	Reduced innate ADCC	CAR specificity
Gene	FCAR	-71.1	IgA receptor	Reduced phagocytic signaling	Reduced variability
Gene	TYROBP	-463.82	innate adaptor	Reduced NK/myeloid signaling	Reduced innate activation
Gene	LILRA5	-56.38	leukocyte receptor	Reduced innate immune signaling	Improved specificity
Gene	CYBB	-155.45	oxidative burst	Reduced ROS generation	Lower inflammation
Gene	FPR1	-59.74	neutrophil chemotaxis	Reduced granulocyte signaling	Reduced innate trafficking
Gene	S100A12	-578.78	inflammatory alarmin	reduced neutrophil inflammation	Lower CRS priming
Gene	FOS	-127.02	early TF	Reduced stress signaling	Controlled activation
Gene	DUSP1	-85.07	MAPK regulator	Reduced inflammatory tone	Functional stability

#### Genes associated with cellular proliferation

These include MKI67 and BUB1, mediating cell cycle progression and active clonal expansion during ex vivo manufacturing.

#### Genes associated with cytokine responsiveness

This showed upregulation of IL2RA, IL12RB2, and CISH, suggesting strong sensitivity to cytokine stimulation, particularly IL-2 family signaling pathways.

#### Genes involved in the effector differentiation

These include IRF4 and BATF3. These transcription factors are known regulators of T-cell activation strength, functional programming, and persistence potential.

#### Genes associated with metabolic fitness

This is represented by PHGDH and PSAT1 demonstrating upregulation of serine biosynthesis pathways required for nucleotide production and cellular growth. Increased expression of LIF further suggests activation of survival signaling pathways that may contribute to CAR-T persistence following infusion.

### Significantly downregulated genes in CAR-T product

Significantly downregulated top ten genes in the CAR-T product in comparison to leukapheresis product were as follows (**Figure 2**):

#### Genes associated with the innate immunity

These include CD14, FCGR3A/B, FCAR, TYROBP, LILRA5, CYBB, FPR1, and S100A12. Their reduction indicates depletion of monocytes, neutrophils, and NK-lineage accessory immune cells present in leukapheresis material.

#### Genes associated with inflammatory response

These include FOS, DUSP1, and S100A12. Their downregulation is suggestive of lower baseline inflammatory transcriptional activity in the final CAR-T product. Reduction of S100A12, a neutrophil-derived inflammatory alarmin, indicates decreased inflammatory priming that may influence downstream cytokine responses following infusion.

#### Genes involved in the Fc receptor signaling

FCGR3A/B, FCAR and oxidative burst pathway (CYBB) were reduced. This indicates removal of phagocytic and antibody-dependent innate immune effector pathways that are not required for CAR-mediated cytotoxicity.

### Reactome pathway analysis

**Figure 3:**
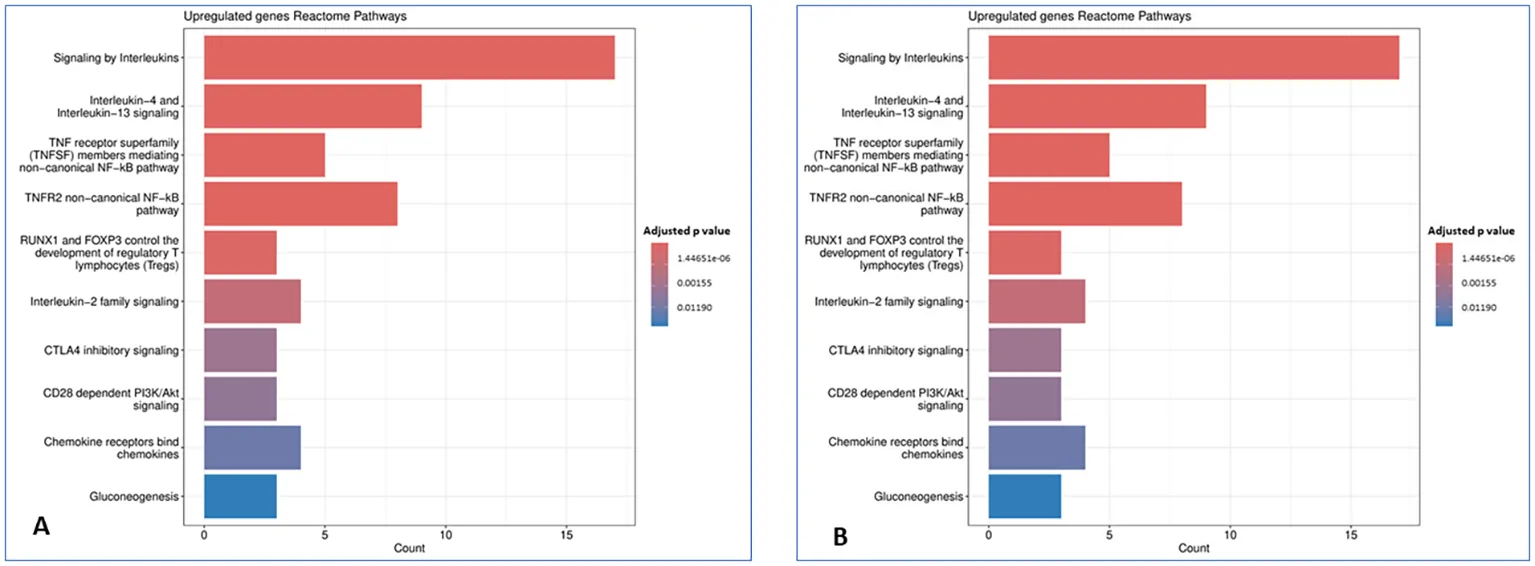
Reactome pathway enrichment analysis of differentially expressed genes identified between manufactured CAR-T bag/product (Stage 2) and leukapheresis (Stage 1) samples. **(A)** Enriched Reactome pathways associated with upregulated genes in the CAR-T bag/product. Bar length represents the number of genes mapped to each pathway, while the color gradient indicates the enrichment P value. **(B)** Enriched Reactome pathways associated with downregulated genes in the CAR-T bag/product. Bar length represents the number of genes mapped to each pathway, and the color gradient denotes the enrichment P value.

Consistent with gene-level findings, Reactome pathway analysis demonstrated enrichment of pathways related to interleukin signaling, IL-2 family signaling, CD28-dependent PI3K/Akt signaling, TNFR2 non-canonical NF-κB signaling, chemokine receptor signaling, and gluconeogenesis (**Figure 3A**). These findings collectively indicate coordinated activation, costimulatory signaling and metabolic adaptation in the CAR-T product (**Table 2**). Enrichment of RUNX1/FOXP3 transcriptional control and CTLA4 inhibitory signaling pathways suggests engagement of physiological regulatory mechanisms that may help maintain controlled activation and prevent excessive immune dysregulation. They also demonstrated downregulation of pathways related to interferon signaling (interferon alpha/beta and interferon gamma signaling), neutrophil degranulation, DAP12 interactions, MyD88 signaling cascade, and Toll-like receptor signaling pathways (TLR1/2/6) (**Figure 3B**). Downregulation of pathways mediating immunoregulatory interactions between lymphoid and non-lymphoid cells further indicates reduced contribution of antigen-presenting cells and innate immune accessory cells in the final product (**Table 2**).

**Table 2:** Reactome pathway analysis in CAR-T product compared to Leukapheresis sample.

Category	Gene/pathway	Pathway regulation	Functional annotation	Interpretation in CAR-T context	Clinical relevance
Pathway	Signaling by Interleukins	Up	cytokine signaling	Activation signaling	CAR-T expansion
Pathway	Interleukin-2 family signaling	Up	IL-2 axis	Proliferation support	Persistence
Pathway	IL-4/IL-13 signaling	Up	cytokine signaling	Differentiation signaling	Immune modulation
Pathway	CD28 PI3K/Akt signaling	Up	Co-stimulation pathway	CAR activation signaling	Expansion kinetics
Pathway	TNFR2 non-canonical NF-kB	Up	survival signaling	Anti-apoptotic signaling	Persistence
Pathway	TNFSF non-canonical NF-kB	Up	immune activation	T-cell survival pathways	Durability
Pathway	RUNX1/FOXP3 regulation	Up	immune regulation	Balanced activation	Controlled toxicity
Pathway	CTLA4 inhibitory signaling	Up	immune checkpoint	Activation feedback control	Reduced exhaustion
Pathway	Chemokine receptor binding	Up	cell trafficking	Tumor homing capacity	Therapeutic targeting
Pathway	Gluconeogenesis	Up	metabolic adaptation	Energy flexibility	Sustained function
Pathway	Immunoregulatory interactions lymphoid-nonlymphoid	Down	APC interaction	Reduced accessory cells	Purified CAR-T product
Pathway	Interferon gamma signaling	Down	inflammatory signaling	Reduced innate activation	Reduced CRS risk
Pathway	Interferon alpha/beta signaling	Down	antiviral response	Lower inflammatory priming	Stable phenotype
Pathway	Interferon signaling	Down	immune activation	Reduced baseline inflammation	Controlled toxicity
Pathway	Signaling by Interleukins (innate components)	Down	inflammatory cytokines	Reduced innate cytokine tone	Improved tolerability
Pathway	Neutrophil degranulation	Down	granulocyte activation	Reduced neutrophil contamination	Reduced inflammation
Pathway	DAP12 interactions	Down	innate signaling	Reduced NK/myeloid activation	Product specificity
Pathway	MyD88: TIRAP cascade	Down	TLR signaling	Reduced pathogen sensing	Controlled activation
Pathway	TLR1:TLR2 cascade	Down	innate sensing	Reduced inflammatory activation	Lower toxicity variability
Pathway	TLR6:TLR2 cascade	Down	innate sensing	Reduced innate immune signaling	Improved consistency

## Discussion

This study represents, to our knowledge, one of the first transcriptomic characterizations of indigenously manufactured approved CAR T-cell products from India compared with matched leukapheresis cells. Our findings highlight both shared biological programs inherent to CAR-T manufacturing and features that may reflect product and population specificity in the context of the India-developed CAR-T.

### Shared transcriptional programs with prior CAR-T studies

The intensive manufacturing process of CAR T-cell therapy remodels the basal transcriptional state by ex vivo activation, genetic modification, and expansion of autologous T-cells. US-based CAR-T studies using bulk and single-cell transcriptomics have revealed significant change of leukapheresis cells towards a proliferative and survival-primed phenotype (7, 8). In preclinical and clinical studies up-regulation of survival and proliferation programs with con-commitment down-regulation of innate inflammatory genes have been linked to persistence of CD19-targeted CAR-T products (9). For example, individuals with long-term CAR-T persistence demonstrated maintenance of TCF7 expression profile (10, 11). This emphasizes that baseline T-cell state and manufacturing-dependent evolution shapes clinical behavior of CAR-T products. In line with these models, our findings display substantial concordance with previously reported molecular programs associated with effective CAR-T activation, proliferation, and effector differentiation. Upregulation of MKI67 and BUB1 reflects a proliferative phenotype consistently reported across CAR-T manufacturing studies, where rapid clonal expansion correlates with therapeutic potency and *in vivo* persistence (12). Cooperative interaction between BATF family transcription factors and IRF4 has been shown to promote effector cytotoxic T-cell differentiation and enhance antitumor activity (13). At the same time, they also counteract exhaustion-associated transcriptional programs (14). Metabolic pathway enrichment through PHGDH and PSAT1 is also consistent with prior reports demonstrating metabolic rewiring toward serine biosynthesis and nucleotide production in activated CAR-T cells (15).

Similarly, the down-regulation of innate inflammatory programs and myeloid-associated genes parallels observations that CAR-T products selectively enrich T lymphocytes and diminish contaminant myeloid programs present in leukapheresis samples. This shift has been linked to improved product purity and reduced pre-activation inflammatory burden. Accordingly, the downregulation of myeloid-associated genes including CD14, FCGR3A, FCGR3B, TYROBP, CYBB and S100A12 is likely attributable to the depletion of monocytes and other non-T-cell populations rather than representing true transcriptional repression within T cells. This is principally supported by studies showing that myeloid signature abundance in leukapheresis associates with poorer manufacturing outcomes and less robust *in vivo* CAR-T expansion (16). To facilitate comparison with previously published studies, the methodological characteristics of major transcriptomic investigations of CD19 CAR-T cell therapy are summarized in **Table 3**. Unlike most published studies, which primarily employed bulk RNA sequencing or single-cell RNA sequencing of pre-manufacture T cells or circulating CAR-T cells after infusion, our study utilized a targeted NanoString immune transcriptomic platform for indigenous CD19 CAR-T bag/product. These differences in CAR construct, sample source, transcriptomic technology and analytical resolution should be considered when comparing molecular signatures across studies. Nevertheless, consistent enrichment of pathways related to T-cell activation, cytotoxicity, persistence and immune regulation across studies supports the biological relevance of current study.

**Table 3:** Methodological comparison of transcriptomic studies evaluating CAR-T cell biology.

Study	CAR-T bag/product	Sample source	Molecular platform	Key objective
Fraietta et al., Nat Med 2018 (8)	CTL019 (tisagenlecleucel)	Leukapheresis samples	Bulk RNA sequencing	Identify transcriptional determinants of clinical response and resistance
Chen et al., Cancer Discovery 2021 (10)	CD19 CAR T	Pre-manufacture T cells	Bulk RNA-seq + single-cell RNA-seq	Define cellular programs associated with long-term CAR-T persistence
Wilson et al., Cancer Discovery 2022 (12)	CD19 CAR-T	Pre- and post-infusion CD19-CAR T cells from blood and bone marrow samples	scRNA-seq + paired TCR sequencing	Track clonal evolution and persistence of effective CAR-T cells
Bai et al., Nature 2024 (7)	CD19 CAR-T	Pre-infusion CAR T cells at baseline and post-infusion peripheral blood CAR-T cells	Single-cell multi-omics (scRNA-seq/TCR profiling)	Characterize durable CAR-T persistence and functional trajectories
Present study (Das et al., 2026)	Talicabtagene autoleucel and Varnimcabtagene autoleucel	Pre-manufacture leukapheresis T cells & Manufactured CAR-T bag/product	NanoString PanCancer Immune Profiling (770 genes)	Longitudinal immune and transcriptomic profiling of indigenous CAR-T therapy

### Distinctive features in the indigenous CAR-T transcriptome

While there is a considerable concordance with established CAR-T profiles, the indigenous CAR-T products exhibit features that may be interpreted through the lens of product design, manufacturing nuances, and population-specific immunobiology. The up-regulation of IL2RA and IL12RB2 supports enrichment of cytokine-responsive effector T cells, consistent with previously described CAR-T activation signatures. Cytokine signaling competence is a recognized determinant of CAR-T expansion and functional persistence. This suggests a nuanced balance between effector expansion and regulatory feedback. The depletion of Fc receptor signaling components (FCGR3A/B, FCAR) highlights reduced antibody-mediated immune activation pathways, reinforcing that cytotoxicity is primarily CAR-driven rather than Fc-mediated. Downregulation of the oxidative burst gene CYBB further confirms removal of phagocytic cell populations capable of generating reactive oxygen species. Reduced expression of early-response transcription regulators FOS and DUSP1 suggests controlled MAPK pathway activation, potentially reflecting optimized ex vivo culture conditions minimizing premature activation-induced dysfunction.

### Contextualizing with clinical experience

The recent regulatory approval of the indigenous Indian CD19 CAR-T therapy NexCAR19 (Talicabtagene autoleucel) underscores the clinical maturation of CAR-T manufacturing capacity in India. In early clinical studies, the product demonstrated objective response rates comparable to those seen in US trials, with notably lower incidence of severe neurologic toxicity and manageable CRS (17). This suggests that transcriptomic features favoring balanced cytokine signaling and controlled inflammatory activation may translate to clinically meaningful safety advantages, particularly in resource-limited settings where intensive supportive care is constrained. US-developed CAR-T products (e.g., tisagenlecleucel, axicabtagene ciloleucel) have been extensively profiled at the transcriptomic level, linking features such as preserved central memory programs and balanced effector/exhaustion signatures with sustained remissions. Shared transcriptional programs involving IRF4–BATF axis activation, proliferative expansion, cytokine responsiveness, and metabolic adaptation support the functional fitness of the CAR-T product. Distinct downregulation of inflammatory alarmins such as S100A12 may represent a potentially favorable molecular characteristic associated with optimized manufacturing conditions. Taken together, the present transcriptional profile demonstrates convergence with established CAR-T activation signatures while highlighting distinctive features suggestive of reduced innate immune contamination and controlled inflammatory tone.

This study represents an exploratory pilot analysis of transcriptomic changes associated with an indigenous CD19 CAR-T bag/product. The limited sample size (four longitudinal paired sample sets) restricts statistical power and the generalizability of the findings. Consequently, the identified molecular signatures should be interpreted as preliminary and hypothesis-generating rather than definitive characteristics of indigenous CAR-T construct. We also acknowledge that several manufacturing-specific variables are manufacturer-dependent and were not uniformly available. Thus, lack of comprehensive manufacturing metadata and clinical correlation analyses present second limitation of the study. Validation in larger, multicenter cohorts with integrated functional and clinical analyses will be necessary to establish the reproducibility and biological significance of these observations. Another limitation of the present study is that transcriptomic analyses were performed on thawed CAR-T bag/products following standard washing procedures. Since CAR-T bag/products were analyzed following cryopreservation, thawing and washing, it is possible that a subset of transcriptional changes, particularly those related to cellular stress and immediate early activation pathways may have been influenced by ex vivo handling. Consequently, the observed molecular signatures should be considered representative of the processed CAR-T bag/product rather than an exact snapshot of the infused cells *in vivo*. The transcriptomic profiles presented in this study should be interpreted in the context of the sample processing workflow. Future studies incorporating fresh infusion products and single-cell multi-omic profiling will help further distinguish intrinsic biological programs from processing-induced transcriptional responses.

To conclude, our findings highlight transcriptomic signatures associated with CAR-T cell manufacturing that provide crucial insights into the biological state of processed CAR-T bag/products. While these signatures are informative and generate mechanistic hypotheses regarding CAR-T function, they should be interpreted in the context of ex vivo sample processing. Furthermore, the comparative analysis of indigenous Indian CAR-T transcriptome against matched leukapheresis support the value of transcriptomic benchmarking in CAR-T development and emphasizes the critical need for integrated multi-omics, longitudinal correlations and standardized analytical frameworks across diverse CAR-T products. Future efforts should aim to correlate these molecular signatures with clinical endpoints to refine manufacturing protocols and personalize CAR-T therapy across global populations.

## Data Availability

The original contributions presented in the study are included in the article/**Supplementary Material**. Further inquiries can be directed to the corresponding author.
