# Supplementary material for: Real-world insights into genomic reprogramming during CAR-T cell manufacturing in India
Source: Front Immunol. 2026 Sep 3;17:1912690. doi: 10.3389/fimmu.2026.1912690 (PMC13582263; doi:10.3389/fimmu.2026.1912690)
Supplement: Supplementary file 1 [file Table1.docx]

**Supplementary Data: Detailed sample processing protocol for PBMC isolation, RNA extraction and n-Counter assay.**

1. **PBMCs (Peripheral blood mononuclear cells) isolation:** PBMCs were isolated using Ficoll-based density gradient centrifugation. Ficoll was layered over the blood. The cells separated according to their densities upon centrifugation. RBCs and granulocytes being denser than Ficoll, pellet at the bottom. Plasma, being the lightest, remained at the top. PBMCs (lymphocytes and monocytes) being lighter than Ficoll but heavier than plasma formed a distinct whitish layer between the two.

**PBMC storage**

Reagent	Reagent Volume	PBMC volume

TRIzol	300ul	200ul

Freezing media	1ml	100ul

The PBMCs were stored in cryovials labelled with specific patient codes and time points and stored at -80℃.

2. **RNA isolation:** RNA isolation was performed using Qiagen QIAamp RNA Blood Mini Kit (Cat. No. 52304): The kit is a column-based RNA isolation technique, where the RNA molecules bind to the silica membrane under certain salt and pH conditions, and the RNA molecules are eluted after washing away the contaminants. The sample is first lysed using buffers with chaotropic salts, which will disrupt hydrogen bonds to inactivate RNases and release the RNA molecules. Chaotropic agents, along with ethanol, facilitate the binding of RNA to the silica membrane, while the contaminants, such as proteins, DNA, and other molecules, pass along with the flow-through. Washing is done to remove any residual impurities. Finally, the RNA molecules are eluted using RNase-free water.
3. **NanoString assay protocol:** All sample preparations were performed according to the manufacturer’s instructions (Nano-String Technologies).

RNA quantity and quality were assessed prior to downstream analysis. Gene expression profiling was performed using the Nano-String nCounter® platform with a targeted immune-oncology panel comprising approximately 750 genes. This panel was designed to capture key biological pathways relevant to CAR T-cell function, including immune activation, cytotoxicity, T-cell differentiation, exhaustion, metabolism, proliferation, and cytokine signaling. Hybridization, post-hybridization processing, and digital counting were performed according to the manufacturer’s instructions.

- ***Hybridization protocol:*** 300 nanograms of total RNA were hybridized overnight with nCounter Reporter (20 μL) probes in the hybridization buffer. An excess of nCounter Capture probes (5 μL) were also placed in the hybridization buffer at 65 °C overnight. After overnight hybridization, the abundance of specific target molecules was quantified using the nCounter digital analyzer.
- ***Scan protocol:*** Individual fluorescent barcodes and target molecules present in each sample were recorded with a CCD camera by performing a high-density scan (600 fields of view).
- ***Description:*** Expression of 770 genes related to components of human nCounter CAR-T panel were analyzed.
- ***Data processing and Statistical Analysis:*** Raw NanoString counts were processed using NanoString’s recommended normalization workflow. Quality control metrics were assessed for all samples. Counts were normalized using internal positive controls and housekeeping genes. Given the paired nature of the study design, differential gene expression analyses were performed by comparing leukapheresis samples with their matched CAR T-cell products.

Further, the fold difference in gene expression was calculated based on sample type and statistical significance was determined using a paired two-tailed t-test within nSolver™ 4.0 analysis software (NanoString nCounter Technologies, Seattle, WA). Background subtraction from raw transcript counts was performed through negative input controls. Normalization to 10 housekeeping genes and differential expression analysis were completed using the advanced analysis software plugin (version 2.0.115). nSolver applied a T-test for calculating the statistical significance (p-value) of the differentially expressed genes (DEGs). Genes were considered differentially expressed if they met the criteria of a FDR adjusted p-value < 0.05 and a fold change > 1.5 (upregulated) or < −1.5 (downregulated). Raw data processing, quality control, and normalization were performed using the for differential expression analysis, a p‐value of ≤0.05, were applied as cut-offs.

**Supplementary Table 1: Total and differential count of leukapheresis products prior to CAR-T cell manufacturing**

| ID | TLC (cells/ul) | Neut% | Ab. Neut (x 10^3^ cells/ul) | Ly% | Ab. Ly (x 10^3^ cells/ul) | Mono% | Ab. Mono (x 10^3^ cells/ul) | Eos% | Ab. Eos (x 10^3^ cells/ul) | Baso% | Ab. Baso (x 10^3^ cells/ul) |
| --- | --- | --- | --- | --- | --- | --- | --- | --- | --- | --- | --- |
|  |  |  |  |  |  |  |  |  |  |  |  |
| CT_01.1 | 27,600 | 26.6 | 7.34 | 31.4 | 8.67 | 1.5 | 0.41 | 0.8 | 0.22 | 3.4 | 0.94 |
| CT_02.1 | 28,710 | 51.2 | 14.71l | 29.9 | 8.59 | 9.9 | 2.83 | 0.1 | 0.03 | 0.6 | 0.17 |
| CT_04.1 | 78,510 | 6.1 | 4.79 | 56 | 44.0 | 19.6 | 15.35 | 0 | 0.0 | 2.2 | 1.74 |
| CT_05.1 | 45, 990 | 45 | 20.72 | 22 | 10.10 | 18.9 | 8.69 | 0.2 | 0.11 | 1.1 | 0.52 |

**Supplementary Table 2: Immunophenotypic characterization of manufactured CAR-T cell bag/product by multiparametric flow cytometry**

| Patient ID | % Lymphocytes (of viable cells) | % T cells (of gated Lymphocytes) | % CD4 cells (of gated Lymphocytes) | % CD8 cells (of gated Lymphocytes) | % CD19+ cells (of gated Lymphocytes) |
| --- | --- | --- | --- | --- | --- |
| CT_01.1 | 99.49 % | 99.42 % | 34.48 % | 62.34 % | 0.00 % |
| CT_01.2 | 99.97 % | 99.38 % | 49.26% | 43.78% | 0.00 % |
| CT_01.3 | 100.00 % | 98.73 % | 88.03 % | 10.16 % | 0.00 % |
| CT_01.3 | 99.86 % | 98.76 % | 27.89 % | 70.23 % | 0.00 % |

**Supplementary Figure 1: Representative gating strategy for immunophenotypic characterization of CAR-T cell bag/product –** Flowcytometric dot plots showing sequential gating on viable CD45⁺ leukocytes to identify CD3⁺ T cells, followed by assessment of CD4⁺ helper T cells and CD8⁺ cytotoxic T cells using fluorochrome-conjugated monoclonal antibodies against CD3, CD4, CD8 and CD45
